# AFM-Based Correlative Microscopy Illuminates Human Pathogens

**DOI:** 10.3389/fcimb.2021.655501

**Published:** 2021-05-07

**Authors:** Supriya V. Bhat, Jared D. W. Price, Tanya E. S. Dahms

**Affiliations:** Department of Chemistry and Biochemistry, University of Regina, Regina, SK, Canada

**Keywords:** atomic force microscopy (AFM), bacteria, correlative microspectroscopy and microscopy, fungi, host-pathogen interaction, microbes, pathogenicity, viruses

## Abstract

Microbes have an arsenal of virulence factors that contribute to their pathogenicity. A number of challenges remain to fully understand disease transmission, fitness landscape, antimicrobial resistance and host heterogeneity. A variety of tools have been used to address diverse aspects of pathogenicity, from molecular host-pathogen interactions to the mechanisms of disease acquisition and transmission. Current gaps in our knowledge include a more direct understanding of host-pathogen interactions, including signaling at interfaces, and direct phenotypic confirmation of pathogenicity. Correlative microscopy has been gaining traction to address the many challenges currently faced in biomedicine, in particular the combination of optical and atomic force microscopy (AFM). AFM, generates high-resolution surface topographical images, and quantifies mechanical properties at the pN scale under physiologically relevant conditions. When combined with optical microscopy, AFM probes pathogen surfaces and their physical and molecular interaction with host cells, while the various modes of optical microscopy view internal cellular responses of the pathogen and host. Here we review the most recent advances in our understanding of pathogens, recent applications of AFM to the field, how correlative AFM-optical microspectroscopy and microscopy have been used to illuminate pathogenicity and how these methods can reach their full potential for studying host-pathogen interactions.

## Introduction

### Relevance of Pathogens

Despite tremendous progress in global health initiatives, the world continues to be confronted by infectious disease, underscoring the need for their survey, prevention, containment and treatment (Bloom and Cadarette, 2019). Humanity and its pathogens share a long and complex history. Pathogens not only threaten our health but represent a significant burden to our health care system, underscoring their research relevance (Bank, 2012; Braden and Tauxe, 2013). Pathogen antimicrobial resistance (AMR) has risen steadily over the past decade, with associated deaths projected to be 10 million by the year 2050, higher than that predicted for cancer (O’Neill, 2016) and associated with a potential global cost of 100 trillion USD. Despite significant AMR research, we are far from understanding the associated mechanisms. Climate change, causing increased environmental fluctuations, has also led to the emergence and re-emergence of pathogens, further reinforcing the need for developing strategies to reduce the burden of infectious diseases (Gudipati et al., 2020).

Vast technological developments over the past-decade in next-generation sequencing and proteomics have helped delineate the genetics of pathogenicity and mechanisms of host-pathogen interactions at the molecular level, respectively (Maljkovic Berry et al., 2020; Rauwane et al., 2020). However, one of the biggest gaps in our understanding of host invasion by pathogens is the accompanying physiological changes, especially during the initial stages of colonization and invasion, which can be viewed and measured using microscopic methods.

### Limitations of Traditional Methods for Studying Pathogens

A variety of methods have been used to study pathogens, the infection process and disease development, including *in vitro* methods (Lob et al., 2017), genetic and molecular studies (Saliba et al., 2017), cell culture (Hudu et al., 2016), animal studies (Margine and Krammer, 2014) and clinical trials (Sambandan and Turcu-Stiolica, 2018). Pathogens have evolved an abundance of mechanisms to invade and overcome host immune responses, an understanding of which is key to disease prevention, diagnosis, control and treatment. The problem is highly complex, such that the variety of pathogenic molecular strategies depend not only on the type of pathogen, but also the host tissue (Sukhithasri et al., 2013). No single approach can characterize the entire complex process, from pathogen encounter to disease development. The parable of the blind men and the elephant can be likened to current molecular platforms, with each technique offering a single restricted view of disease development. To develop a more comprehensive picture we often combine and integrate multiple techniques within a single study.

Our success in studying pathogens is largely tied to our current technological limits. Traditional biochemical techniques require significant sample processing, which not only introduce bias but often represent the average behavior of a large population of cells (Li et al., 2019). Recent computational advances have powered the ‘omics’ era, yielding extensive high-throughput data at the genetic, transcript, protein and metabolic level, along with integrated multi-omics data (Khan et al., 2019). However, analysis of this information-rich omics data is complex, and the associated experimental methods can be expensive and time-consuming. Large scale proteomic interaction studies require multiple protein purification steps that rely on physical and chemical affinity-based principles, with a high probability of false interactions (Minic et al., 2018). Individual cells have unique behaviors which are overlooked by omics studies of large ensemble populations, although single cell multi-omics is expected to be more broadly applied in future (Hu et al., 2018). For instance, medically important phenomenon such as antibiotic resistance and fitness advantages, resulting from spontaneous mutations and favorable for host adaptation and invasion, are acquired by individual cells which then replace the entire unfit population.

Both traditional biochemical assays and omics require additional approaches for validation. For example, (Abhyankar et al., 2019) used proteomics to study bacterial spore integrity and germination physiology, showing a large variation in sporulation and germination kinetics which necessitated fluorescence imaging of individual spore germination, outgrowth and intracellular pH dynamics. Furthermore, pathogen mutational studies are incomplete without phenotypic characterization of individual pathogens *in situ* (Conrad et al., 2018).

## Using AFM to Study Pathogens and Their Behavior

Atomic force microscopy (AFM) was originally developed (Binnig et al., 1986) to characterize semiconductor devices (Alonso and Goldmann, 2003; Ricci and Braga, 2011; Chang et al., 2012; Dufrêne et al., 2017; Krieg et al., 2019), but was recognized for its potential to image non-conducting samples such as delicate biological specimens. Unlike optical and electron microscopy (EM), AFM scans a cantilever-mounted tip over the sample surface, with tip-sample forces used for feedback control of piezoelectric micropositioners required to contour the biological specimen. Such forces are a complex weave of physical, chemical and biological interactions, which can be quantified by sensing the resultant cantilever deflection (Krieg et al., 2019).

AFM has become a powerful technique for probing live biological systems, providing information on diverse parameters such as morphology, surface ultrastructure and nano-mechanical properties (force nanoscopy) at the sub-molecular level (Liang et al., 2020). Notable advantages of AFM over the widely used EM techniques include expedient and simple sample preparation, live cell imaging under a controlled physical environment and a high signal-to-noise ratio (S/N). AFM offers high content data for characterizing cell surfaces (Bhat et al., 2015), membranes and envelopes (Viljoen et al., 2020), cell division (Formosa et al., 2013; Bhat et al., 2018a) and adhesion (Mathelié-Guinlet et al., 2019). Systems studied by AFM range from viruses (Chen et al., 2013) to embryonic tissue (Chevalier et al., 2016; Thompson et al., 2019), parasites (Sinha et al., 2015; Perez-Guaita et al., 2018; Ma et al., 2020) and other pathogens (Alsteens et al., 2009; Deupree and Schoenfisch, 2009; Alsteens et al., 2013).

Even though AFM is a powerful technique offering exceptionally high resolution, it has many inherent drawbacks; it is a surface scanning technique therefore providing minimal information on internal structures, samples need to be immobilized, and it has a small field of view with limited vertical range (Dufrêne, 2002). AFM is highly sensitive to sound and vibrational noise, is subjected to thermal drift and limited in its ability to track molecular interactions in real time (Zhou et al., 2017). Interaction of the probe with delicate biological samples can also displace and potentially damage the samples during force nanoscopy. Some of these limitations have been addressed by combining AFM data with that from other microscopic methods such as fluorescence and electron microscopy. With the development of high-speed AFM it is now possible to obtain information on molecular level dynamics (Ando, 2018).

### Quantifying Pathogen Adhesion

AFM is an indispensable tool for understanding bacterial adhesion (Beaussart et al., 2014), a first step of pathogenic invasion, including surface sensing and attachment to the host cell surface (Bukrinsky et al., 2020). Pathogens sense a change in their physico-chemical environment, and given appropriate conditions can dramatically alter their phenotype and physiology in preparation for attachment (Ellison et al., 2019). In pathogenic *Escherichia coli*, and many other bacteria, surface sensing and attachment is mediated by long appendages called pili and fimbriae (Ellison et al., 2017). Following attachment, *E. coli* must subvert host responses by the secretion of protein factors, eventually resulting in a coordinated invasion of host cells and efficient colonization. Gene knockout studies have elucidated the role of pili in adhesion to abiotic surfaces (Proft and Baker, 2009; Danne and Dramsi, 2012; Maier and Wong, 2015; Craig et al., 2019; Lukaszczyk et al., 2019), however proper functional characterization requires high resolution phenotyping. Pili, made up of the pilin proteins, are also known to be involved in pathogenicity, horizontal gene transfer, surface sensing, motility, biofilm formation, swarming, quorum sensing and microcolony formation (Berne et al., 2015; Ellison et al., 2019). AFM has been an indispensable tool for characterizing fimbrial adhesion mechanisms, and combined with molecular and other high-resolution microscopy techniques for characterizing the structural and functional characteristics of pili. For example, Hansmeier et al. (2017) detail the ultrastructural features of the entire “adhesiome” of *Salmonella enterica*, including many cryptic and non-fimbrial adhesins. AFM force measurements show how fluid drag forces enhance binding of FimH protein to mannose, facilitating *E. coli*-intestinal interaction and infection (Yakovenko et al., 2008). The spring-like nature of FimH was also demonstrated by AFM cantilevers, capable of stretching the Type I fimbriae far beyond its original length (Forero et al., 2006). Beaussart et al. (2016) were able to use AFM-based force nanoscopy to evaluate the anti-adhesive properties of novel multivalent mannofullerines and their potential to prevent uropathogenic *E. coli* adhesion to host cells. Fluorescence microscopy has been extensively used to understand the real-time temporal and physical perturbations of adhesion to surfaces facilitated by fimbriae (Ellison et al., 2019), underscoring the future potential of correlative fluorescence-AFM to address similar research questions.

### Quantifying Host-Pathogen Interactions

Complementary to biochemical assays and omics methods, the strength of AFM lies in its ability to analyze and nano-manipulate single cells with high spatial-temporal resolution (reviewed in (Li et al., 2019). Single-cell analysis is crucial for understanding the adhesion that drives the first-steps of host-pathogen interactions (Beaussart and El-Kirat-Chatel, 2019). Many molecular methods have been used to study host-pathogen surface interactions, but only AFM can localize and quantify those interactions at the nm and pN scale, respectively. For example, Formosa-Dague et al. (2016) characterized the molecular interactive forces between *Staphylococcus aureus* and corneocytes using AFM cantilevers linked with single bacteria, showing a high adhesion force (500 nN) between the two, originating from bacterial surface adhesins and specific ligands on the corneocyte surface. Using a combination of ﻿AFM, Raman micro-spectroscopy and scanning EM, Alvarado-Gomez et al. (2018) quantified the physicochemical properties of single- and multi-species biofilms consisting of antibiotic resistant strains of *S. aureus* and *Pseudomonas aeruginosa* isolated from infected chronic wounds, and their adhesion forces to glass surfaces. The inverse relationship between viability and adhesion for the two species in the context of the multi-species biofilm, and the shift from a single to multilayer biofilm for the mixed-species underscores the complexity of clinical biofilms, which must be considered when developing treatment options. Pathogen adhesion to abiotic surfaces such as catheters and other medical instruments is a major source of secondary infections in hospitals (Buetti et al., 2018). The adhesive forces of *Caulobacter cresentus* (Sprecher et al., 2017), *E. coli*, *Streptococcus pyogenes* (Potthoff et al., 2015), and variety of plant pathogens (Mittelviefhaus et al., 2019) to abiotic surfaces have been successfully probed using single-cell force spectroscopy (SCFS) and fluidic force microscopy (FluidFM) (Potthoff et al., 2015). The ability of AFM to measure nano-mechanics also has significant biomedical applications, exemplified by the role of *Klebsiella pneumoniae* exo-polysaccharide capsule in biofilm formation and the underlying bio-physical mechanism. Theoretical modeling of the associated AFM mechanical data indicate capsule structural organization and type 3 fimbriae are important for adhesion, and thereby biofilm formation (Wang et al., 2015).

### Role of the Cell Wall in Pathogenicity

The cell wall of fungal and bacterial pathogens is the first point of contact and most important structure for maintaining viability, cell architecture, and protection from the external environment, including antibiotics and host immune factors (Meroueh et al., 2006; Egan et al., 2017). We do not yet fully understand how the pathogen cell wall responds to varying environmental and host stress factors, and for fungal pathogens, we are still in the early stages of examining the mechanism of host sensing and attachment. The fungal cell wall is crucial for virulence, and AFM is a valuable tool to image its ultrastructure at the nano-scale while measuring its viscoelastic properties, adhesion forces to host, and even its molecular composition using functionalized AFM tips (Beaussart et al., 2012; Formosa et al., 2013; Dufrêne et al., 2017; Li et al., 2017; Shahina et al., 2018; Turner et al., 2018). El-Kirat-Chatel et al. (2015) used AFM force nanoscopy to demonstrate how Epa6 adhesins on the cell surface of *Candida glabrata* help mediate adhesion to abiotic surfaces through strong hydrophobic interactions. Valotteau et al. (2019) subsequently used an elegant combination of genetics and AFM mediated single-cell force spectroscopy (SCFS) to characterize the role of Epa adhesins in fungal adhesion by measuring the adhesion forces of a series of *C. glabrata* Epa adhesin knockout mutants to abiotic surfaces.

### Characterizing Viral Infectivity

Another remarkable feature of AFM is its ability to make precise atomic-scale measurements of living biological specimens. Naturally AFM has been frequently used for making precise measurements of viral capsids (de Pablo and Schaap, 2019), probing the mechanics of capsid-genome interactions (Yang et al., 2018), imaging morphological changes that drive maturation (Pang et al., 2013), and conducting nano-manipulations (Li et al., 2014) to understand structure-function. For example, AFM has been used to examine viral response to shell disruption induced by mechanical stress (de Martinez-Martin et al., 2012; Marchetti et al., 2016; Pablo, 2018; Roos, 2018), membrane fusion (Godefroy et al., 2014; Hilsch et al., 2014), viral genetic material at high-resolution (Ares et al., 2016), protein self-assembly with high-speed AFM (HS-AFM) (Nievergelt et al., 2018) and capsid disassembly (Rankovic et al., 2017). The scanning electrochemical microscopy (SECM) mode of AFM has been used to image viral particles labeled with redox antibodies, producing high-resolution topography images upon which labeled proteins can be selectively localized from the electrochemical current image (Nault et al., 2015). While it is a powerful method, SECM remains a highly specialized technique which requires samples with electrochemical properties, and so it has not been broadly applied to live cells (Izquierdo et al., 2018).

Certain pathogen processes, such as adhesion to host cells, lead to biochemical and physical changes at the molecular surface of both the host and pathogen, which are eventually transmitted intracellularly through signal-transduction to determine cell physiology (Kok et al., 2020). Since AFM is restricted to surface measurements, its physical integration with other microscopes allows insight into intracellular events that accompany or are impacted by surface alterations.

## Understanding Microbial Pathogenicity, a Correlative Microscopy Approach

**Figure 1:**
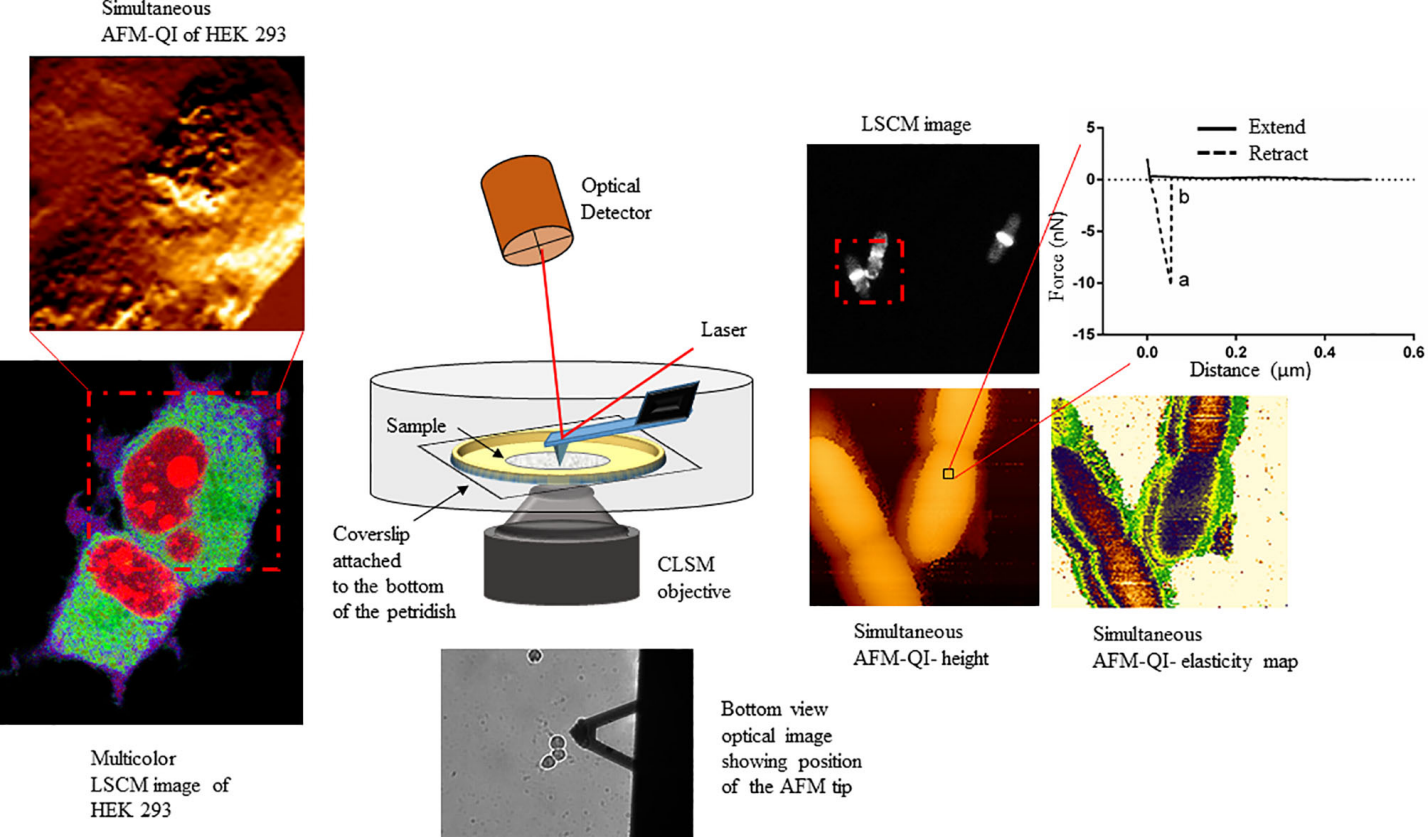
Schematic showing the multiplexed data arising from correlative atomic force-confocal microscopy. AFM in quantitative imaging (QI™) mode quantifies elasticity, adhesion (extracted from nanoscopy curves, top right) and surface topography, while confocal can localize multiple fluorescently labeled molecules within the sample (bottom left). Simultaneous imaging can be conducted in live, physiologically relevant conditions in real-time. This method can be easily extrapolated to characterizing human-pathogen interactions, in the presence or absence of therapeutics, at the single-cell level. This figure has been reproduced from Bhat et al. (2018b) with permission from *Scientific Reports*.

The field of correlative microscopy seeks to bridge the gap between different microscopic techniques, for example by extending field of view (FOV) ranges or probing different cellular regions. AFM, with its unprecedented surface resolution, when combined with optical microscopy expands the FOV range from nm to mm to view the smallest molecules to the entire cell (**Figure 1**), colony or tissue, offering a comprehensive view of host-pathogen interactions, from the outermost surface to the deepest cellular compartment, with detailed insight into pathogenicity.

### Tracking Cell Wall Changes With Correlative AFM-Microspectroscopy

A relatively new correlative method uses synchrotron-based infrared nanospectroscopy (SINS) to illuminate an AFM tip for the dual purpose of nanoscale imaging while using near-field infrared spectroscopy to report on local chemical groups just nanometers below the tip (Bakir et al., 2019). SINS of the opportunistic pathogen *Aspergillus nidulans* and its mutants lacking the sugar galactofuranose, involved in pathogenicity and cell wall function, showed changes in cell wall biochemistry and surface roughness, best attributed to the cell wall integrity pathway (Bakir et al., 2019). Kochan et al. (2018) used a similar approach to monitor dynamic changes in the cell wall of six bacterial species, including pathogenic *S. aureus* and *E. coli*, during cell division, showing changes to complex carbohydrate and phosphodiester groups, including peptidoglycans and teichoic acid, and enabling discrimination of Gram-positive and Gram-negative cell wall components. Using a single wavenumber, AFM-IR maps have been generated for *Plasmodium falciparum* subcellular structures in the context of a red blood cell infection, during intraerythrocytic development, eliminating the need for EM sectioning (Perez-Guaita et al., 2018). Since the temporal resolution of these methods rely on the AFM raster scan, the addition of high-speed AFM scanners (Uchihashi and Scheuring, 2018) to SINS will facilitate much faster image collection rates for viewing dynamic processes.

### Correlative AFM-Optical Microscopy Shines a Light on Microbial Pathogens

**Figure 2:**
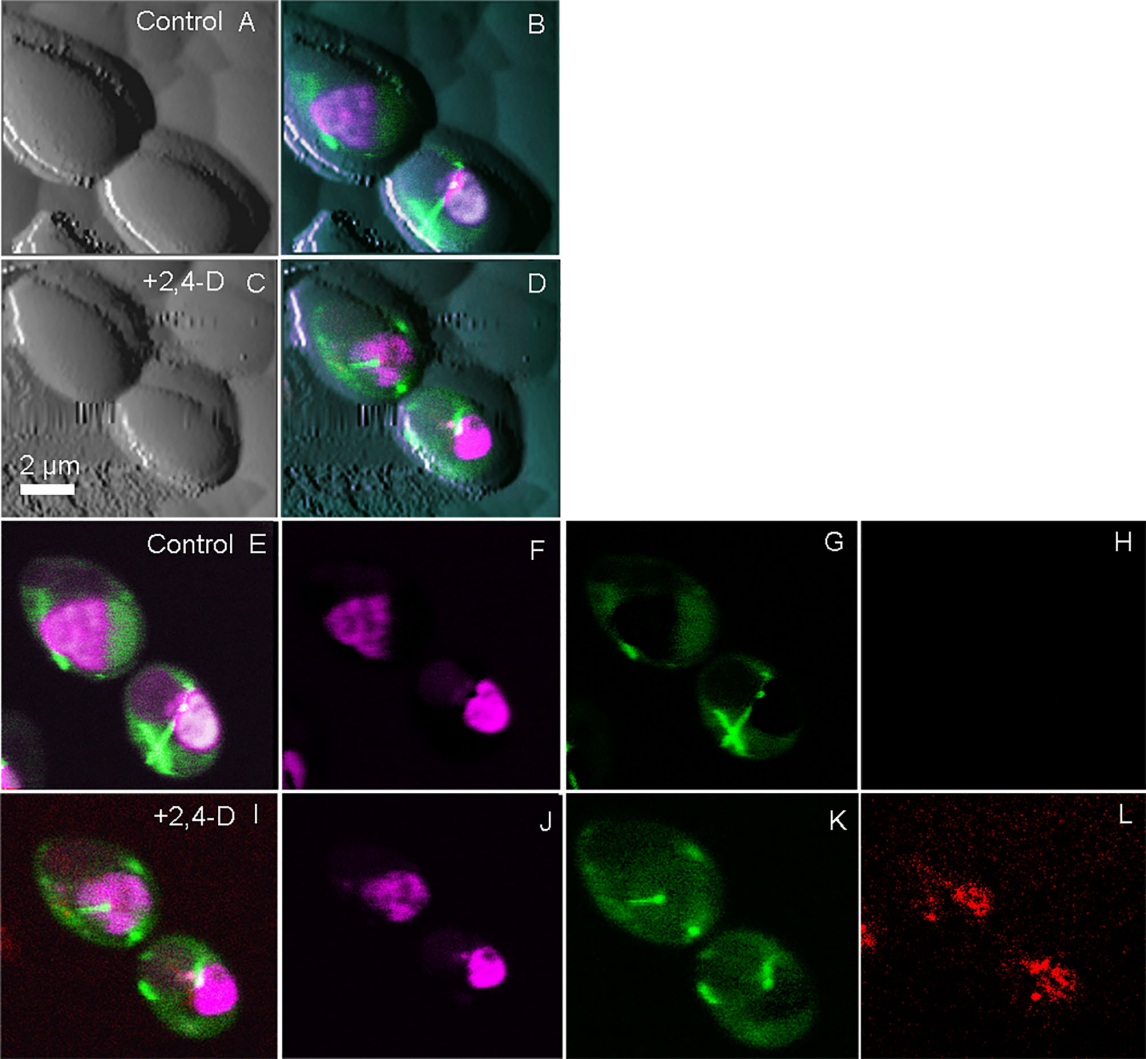
Simultaneously collected correlative AFM-confocal images of the human fungal pathogen *C. albicans*. Shown is the localization of tubulin2-GFP (green), histone protein B-RFP (purple) and reactive oxygen species (Cell-ROX-deep red, red) following exposure to the herbicide 2,4-D, which chemically induced oxidative stress. Tricolored confocal images are overlaid onto AFM images before and after the induction of ROS **(A–D)**. Confocal images in the absence of 2,4-D **(E–H)** are compared with those 5 min after 2,4-D exposure **(I–L)** showing ROS induction **(L)**. **(E, I)** are tricolor merged confocal images; **(F, J)** shows only RFP, G and K show only GFP and **(H, L)** show only ROS. The figure has been reproduced from Bhat et al. (2018b) with permission from *Scientific Reports*.

Optical microscopy is one of the few techniques capable of probing intracellular structures and molecular interactions in live cells at video rate. Conventional light microscopy is diffraction limited, with a maximal resolving power (~ 0.2 μm), larger than the size of most subcellular structures, and only achievable under optimal conditions. Fluorescence was added to the optical microscopy suite in the early 1900s, offering enhanced contrast, single protein specificity and sensitivity (Perfetto et al., 2004). Wide field fluorescence microscopy can resolve single molecules that are sufficiently separated, but suffers from low signal to noise from the detection of out of focus light, therefore limiting contrast. Confocal microscopy solves this problem by combining focused laser excitation with pinhole (a small aperture) detection to reject out-of-focus light and optimize fluorescence contrast [reviewed in (Sanderson et al., 2014)]. Laser scanning confocal microscopy (LSCM) is possibly the most popular technique of the past decade, in part based on the development of powerful and sophisticated modalities, including recently developed super-resolution microscopy (Vangindertael et al., 2018). Bhat et al. demonstrated the utility of correlative AFM-LSCM to simultaneously acquire multiplexed data (**Figure 1**), including surface roughness, adhesion, elasticity, localization and dynamics of intracellular molecules in real-time for live *C. albicans*, *E. coli* and HEK cells during induced oxidative stress (Bhat et al., 2018b). This study details the common and differential responses of two pathogens (**Figure 2**) and human cells to external stress, offering the possibility of studying live host-pathogen interactions in real time. Correlative AFM-confocal is applicable to problems that have been explored by AFM and confocal in tandem. For example, Peters et al. (2012) used AFM to quantify the interaction of two leading nosocomial pathogens, *S. aureus* and *C. albicans*, and confocal to localize the *C. albicans* surface adhesin protein Als3p to identify its role in mediating *S. aureus* adhesion to fungal hyphae. Initial pathogen adhesion and host-pathogen interaction, key to successful host invasion, has been successfully captured by correlative AFM-fluorescence (El-Kirat-Chatel and Dufreîne, 2012). El-Kirat-Chatel and Dufrêne observed various stages of the *C. albicans* infection process, including initial contact with host macrophages, pathogen internalization, intra-cellular hyphal growth and eventual pathogen escape.

Both AFM and confocal microscopy have been instrumental in understanding biofilm phenotype, ultrastructure and behavior. Control and treatment of biofilm forming pathogens is a serious clinical challenge based on their ability to resist commonly prescribed antimicrobials (Qin et al., 2009). Gonçalves et al. (2017) characterized the morphological and biochemical changes to *C. albicans* biofilms during exposure to the antimicrobial defensin Psd1, using AFM to follow membrane disruptions and leakage in tandem with confocal to track *C. albicans* viability within the biofilms. Ahmed et al. (2016) quantified the anti-biofilm activity of chlorohexidine conjugated gold nanoparticles against *K. pneumoniae* ATCC13882 and clinical isolates, using AFM to characterize the biofilm surface ultrastructure and confirm nanoparticle morphology while fluorescence microscopy visualized biofilm eradication during the chlorohexidine treatment. Such research questions are well served using a correlative microscopy approach.

### Correlative AFM-SMLM Defines Pathogenicity at the Single Molecule Level

Techniques such as stochastic optical reconstruction microscopy (STORM), photoactivated localization microscopy (PALM), and other forms of single-molecule localization microscopy (SMLM) can be used to visualize individual proteins in live cells by virtue of highly specific dyes that can be switched on and off (Zhou et al., 2020). The associated images are collected in a time-resolved fashion and the image frames are later reconstructed using specialized software to create an image that is not diffraction limited (Hirvonen and Cox, 2018). A major limitation of SMLM relates to context – the lack of phenotypic image on which to map the data can be likened to gazing into a starry night sky without a cosmic map, making it nearly impossible to understand the relationship of each star to the other. AFM is a prime choice for contextualizing such data, by providing a topographic map on which to localize the visualized proteins, especially since it is compatible with live samples and matches the resolving power of SMLM. Odermatt et al. (2015) generated the first correlated AFM-PALM images of cells, along with methods to image bacteria in aqueous media. More recently Zhou et al. (2020) localized membrane cytoskeletal complexes, offering the potential to further delve into membrane structure and its associated domains as a function of pathogenicity. Understanding pathogen-host interactions facilitated by ligand-receptor binding and signal trafficking is key for novel drug discovery. Pathogen adhesion triggers a variety of signaling cascades that operate within different time frames, including recruitment of various classes of membrane molecules that play a role in eliciting host immune responses. Ciczora et al. (2019) characterized and quantified the early stages of *Yersinia pseudotuberculosis* adhesion to HeLa cells to examine the recruitment of glycosylphosphatidylinositol (GPI)-anchored protein domains to the binding site at the bacterial cell surface, with concomitant cytoskeletal re-arrangements. Bacteria-functionalized AFM cantilevers delivered bacteria to the HeLa surface where a suite of fluorescence microscopy techniques (TIRF, STORM, PALM) were used to view GPI dynamics during adhesion and entry, comparing the concomitant actin remodeling and cell signaling activated during pathogenic adhesion versus that during internalization (Ciczora et al., 2019).

Pathogens commonly target the cell cytoskeleton for the purposes of attachment, entry, and movement within and between cells (Gruenheid and Finlay, 2003; Zihni et al., 2014). Correlative STED/STORM-AFM has been frequently used to correlate membrane physical properties with cytoskeletal structure and dynamics, in which AFM produces the high-resolution topographical information upon which STED can localize labeled actin/microtubule dynamics during an external stimulus (Chacko et al., 2013; Curry et al., 2017). A relatively new correlative technique developed by Janel et al. (2017) combines AFM with superresolution light microscopy (STED) and electron microscopy, named CLAFEM. This technique has been used to observe *Yersinia pseudotuberculosis* infection in Ptk2 cells, revealing its precise location along with that of host organelles, and the mechanical properties of each (Janel et al., 2019). In a recent study, Nelsen et al. (2020) ﻿present a new correlative AFM-volumetric light sheet fluorescence microscope for which they provide proof-of-principle by visualizing lysosome trafficking, vimentin nuclear caging, and actin dynamics in HeLa cells, as well as demonstrating how the force exerted by a macrophage during phagocytosis can be correlated with local actin dynamics at high spatial-temporal resolution. Taken together, these studies demonstrate the enormous future potential of correlative super-resolution fluorescence-AFM to uncover pathogen-host dynamics.

### Understanding Virus-Host Interactions by Correlative Microscopy

**Figure 3:**
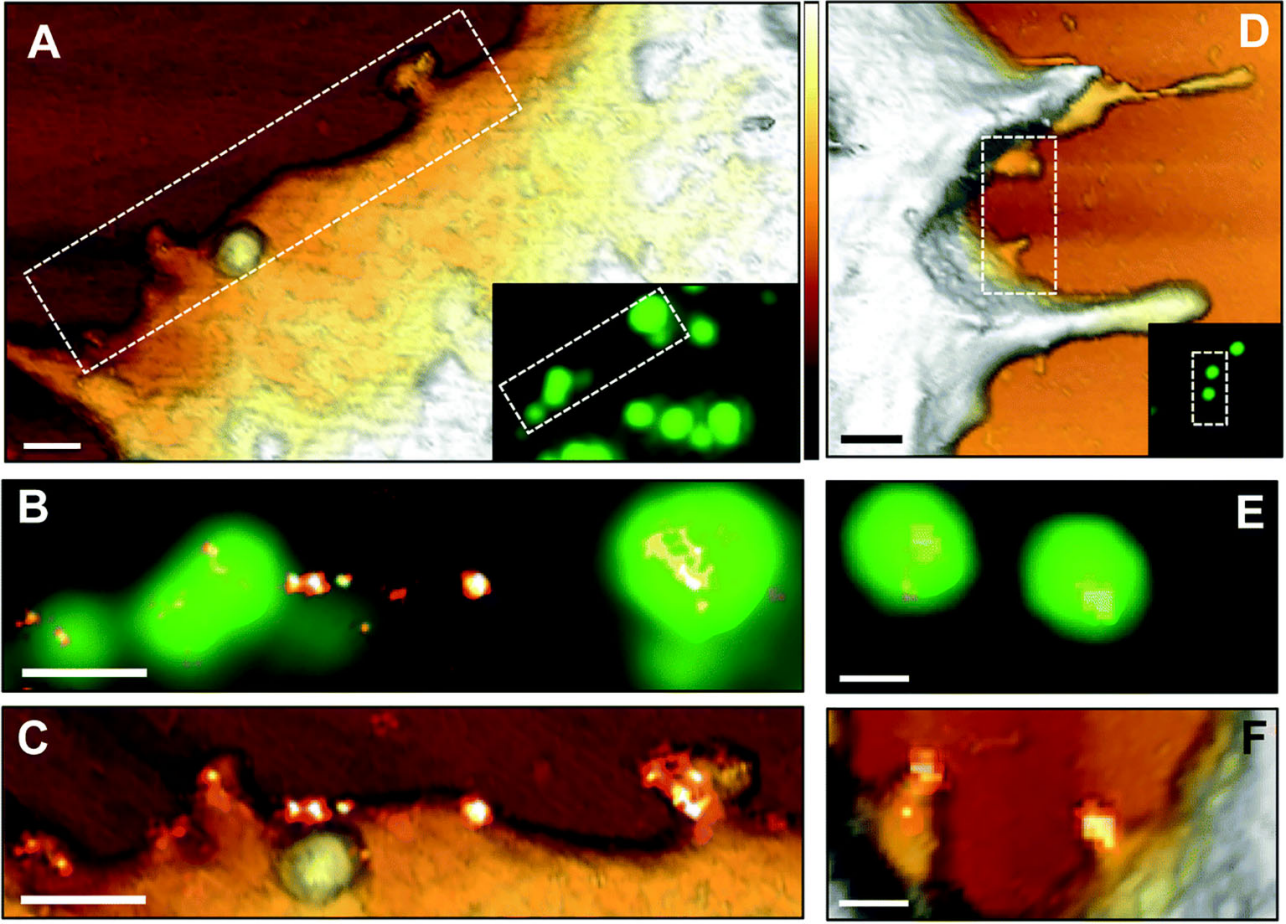
AFM correlative-dSTORM super resolution microscopy demonstrates CD9 recruitment at HIV-1 budding sites. HIV-1 Gag-GFP expressing HeLa cells were labelled with anti-CD9-Alexa-647 and imaged by AFM **(A, D)**, conventional fluorescence **(B, E)** and dSTORM **(C, F)**. Dotted rectangles on the AFM images **(A, D)** indicate areas examined at higher resolution by AFM **(C, F)** and by fluorescence for the Gag-GFP signal **(B, E)** and the AFM images are overlaid with the reconstructed dSTORM image of tetraspanin CD9 **(C, F)**. Scale bars are 500 nm **(A, D)** or 200 nm **(B, C, E, F)**. The Z scale colorized in A and D is 300 nm, with the lightest regions representing the highest points. The figure has been reproduced from Dahmane et al. (2019) with permission from *The Royal Society of Chemistry* (https://pubs.rsc.org/en/content/articlelanding/2019/nr/c8nr07269h#!divAbstract).

There is already significant information on the life cycle of viruses in host cells, which has been accelerated by the SARS-CoV-2 pandemic. However, the regulatory mechanisms involved in the initial surface sensing and interaction are not well understood, mainly limited by the lack of techniques available to decipher the molecular interactions between the virus and host surface receptors (Alsteens et al., 2017). AFM-based correlative microscopy is especially promising for elucidating viral-host binding events based on its ability to simultaneously examine surfaces at high-resolution and intracellular events. For example, Newton et al. (2017) used AFM tips functionalized with the MuHV-4 viral particle to elucidate the multivalent interactions that allow the virus to strengthen its attachment to the host surface, while simultaneously observing the associated intracellular events. A year later, Delguste et al. (2018) used correlative AFM-confocal to record the mechanical events and adhesion forces during the viral infection process, including surface receptor-viral interactions. Yang et al. (2018) demonstrate the utility of using SMLM to characterize HIV-1 assembly events at the plasma membrane by elucidating the role of viral RNA in mediating the assembly of Gag proteins into viral particles using correlative AFM-photoactivated localization microscopy (PALM) with single-particle tracking. Finally, correlative dSTORM-AFM was used to localize and map the tetraspanin proteins, revealing the HIV-1 assembly and release mechanism from host cells (Dahmane et al., 2019) (**Figure 3**).

### Challenges and Limitations of Correlative Microscopy

One of the major drawbacks of correlative AFM-fluorescence systems is the cross-talk resulting from the AFM laser beam, passing through and beside the cantilever, entering the optical path (Ebenstein et al., 2002; Kassies et al., 2005). Several commercially available instruments use infrared super-luminescent diodes (SLD) that reduce emission at most wavelengths, allowing visualization of the probe while maintaining strong fluorescence signal to noise. Though SLDs reduce noise and laser interference to some extent, incorporation of a narrow band filter is required for complete removal of laser interference when doing sensitive optical measurements. Unfortunately, these filters are difficult to incorporate and are expensive. Fernandes et al. (2020) were able to achieve cross-talk free correlative AFM-confocal microscopy by defining experimental parameters such as tip geometry, material, and metallic coating at the cantilever backside, as well as cantilever stiffness. The authors showed accurate real-time acquisition and two-dimensional mapping of interaction force, fluorescence lifetime and intensity, characterizing morphology (AFM) and local viscosity (FLIM) of gel and fluid phases for supported lipid model membranes (Fernandes et al., 2020).

Another major issue with integrated AFM-LSCM is the coupling of vibrational and acoustic noise caused by LSCM rotating parts and laser fan, respectively, which can disrupt the extremely sensitive AFM measurements (Bhat et al. 2018b). To address this issue, Trache and Lim (2009) isolated all sources of vibration and acoustic noise by mounting the confocal scanner on a silicone damping pad and by isolating other vibrating parts such as camera fans by physically separating them from the microscope body. Nano manipulation experiments are time consuming and can last for hours, so photo bleaching can be a significant problem. This issue has been overcome using extremely sensitive cameras that allow the minimum usage of laser intensity for excitation (Kondra et al., 2009).

The recently developed super-resolution techniques have surpassed the conventional diffraction limit down to a few nm, however they suffer several drawbacks. Super-resolution techniques are based on the use of specialized dyes that must provide increased brightness, specificity and stability, but photo-switchable dyes can be toxic, along with the typical undesirable effects of large fluorophores and fluorescent tags that can impact protein folding and function. Even though small molecular weight fluorophores have been widely used to study intracellular dynamics, covalently bound fluorophores can result in mis-folded protein aggregates (Cosentino et al., 2019). The reconstruction of simultaneously collected AFM and PALM/STORM/STED images also suffer image overlap artefacts (Gómez-Varela et al., 2020). Superresolution imaging requires a buffer containing an enzymatic oxygen scavenger to enable photoswitching of the dye molecules; however oxygen scavengers are known to interfere with AFM cantilevers. Towards overcoming this issue, Hirvonen and Cox (2018) studied a new red cyanine dye, iFluor-647, which has superior brightness and blinking properties even in the absence of the enzymatic oxygen scavenger. Since many of the limitations associated with confocal and superresolution techniques have been addressed, AFM-correlated super-resolution techniques are more powerful than ever for characterizing host-pathogen interactions.

## Conclusions and Future Perspectives

AFM has provided unprecedented detail on host-pathogen interactions, single cell nano-manipulation and mechanics, and viral-binding events. The power of AFM is enhanced when used in combination with other techniques capable of biochemical mapping, and is complementary to traditional molecular genetics methods to study pathogens. Minimal sample preparation coupled with the ability to image at the nm-scale and measure pN forces in virtually any environment have made AFM irreplaceable in biomedicine. The paradigm of our scientific approach is rapidly changing, currently reflecting the ever-evolving instrumentation and multidisciplinary research methods. In this context, AFM-based correlative microscopy is making truly significant contributions to a variety of fields, shedding light on pathogen behavior, properties and pathogen-host interactions. Correlative AFM-super resolution microscopy has already been applied to receptor-ligand mapping, mechanotransduction studies and single-cell nanomanipulation, offering information that is highly complementary to molecular and genetic studies. In future, AFM and correlative AFM-optical microscopy will continue to be used to tackle a wide variety of problems. These non-invasive optical imaging techniques, enabled by ever more sophisticated fluorescent probes, allow the investigation of almost any cellular event, with single cell AFM-based correlative microscopy able to measure fine-scale pathogen-host interactions. In conclusion, correlative AFM-optical microscopy represents a series of established high content methods that offer enormous potential to expand our understanding of pathogen processes and proper exploitation of these techniques has unlimited application in biomedicine and pathogen research.

## Author Contributions

JP conducted the initial literature searches and generated a preliminary outline. SB established the trajectory of the review in consultation with TD, and wrote a first draft and edited along with TD. All authors contributed to the article and approved the submitted version.

## Funding

SB was funded by a Natural Science and Engineering Research Council of Canada Discovery grant to TD (RGPIN-2018-06649).

## Conflict of Interest

The authors declare that the research was conducted in the absence of any commercial or financial relationships that could be construed as a potential conflict of interest.
